# Cinnoline Scaffold—A Molecular Heart of Medicinal Chemistry?

**DOI:** 10.3390/molecules24122271

**Published:** 2019-06-18

**Authors:** Marta Szumilak, Andrzej Stanczak

**Affiliations:** 1 Department of Hospital Pharmacy, Faculty of Pharmacy, Medical University of Lodz, 1 Muszynskiego Street, 90-151 Lodz, Poland; 2 Department of Applied Pharmacy, Faculty of Pharmacy, Medical University of Lodz, 1 Muszynskiego Street, 90-151 Lodz, Poland; andrzej.stanczak@umed.lodz.pl

**Keywords:** cinnoline, biological activity, heterocyclic compounds

## Abstract

The cinnoline nucleus is a very important bicyclic heterocycle that is used as the structural subunit of many compounds with interesting pharmaceutical properties. Cinnoline derivatives exhibit broad spectrum of pharmacological activities such as antibacterial, antifungal, antimalarial, anti-inflammatory, analgesic, anxiolytic and antitumor activities. Some of them are under evaluation in clinical trials. In the present review, we have compiled studies focused on the biological properties of cinnoline derivatives conducted by many research groups worldwide between 2005 and 2019. Comprehensive and target oriented information clearly indicate that the development of cinnoline based molecules constitute a significant contribution to the identification of lead compounds with optimized pharmacodynamic and pharmacokinetic properties.

## 1. Introduction

**Figure 1:**
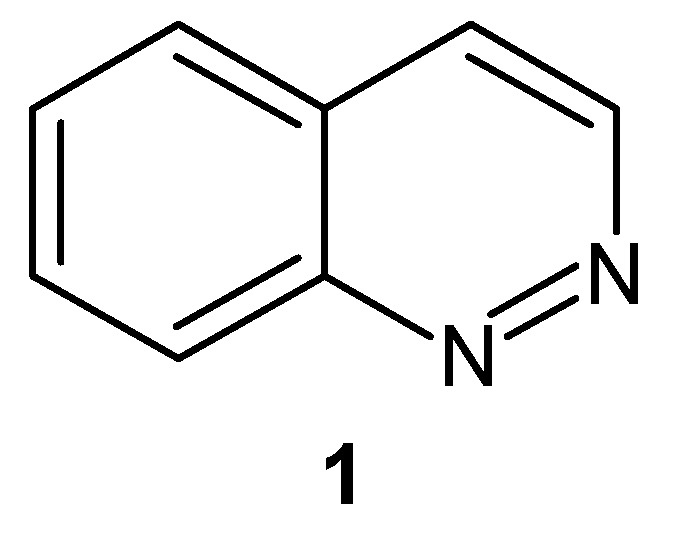
Structure of the cinnoline ring system.

Cinnoline (1,2-benzodiazine) **1**, depicted in Figure 1, is present in many compounds of considerable pharmacological and chemical importance [1]. It is six-membered ring system with two nitrogen atoms, an isosteric relative to either quinoline or isoquinoline and isomeric with phthalazine [1,2].

**Figure 2:**
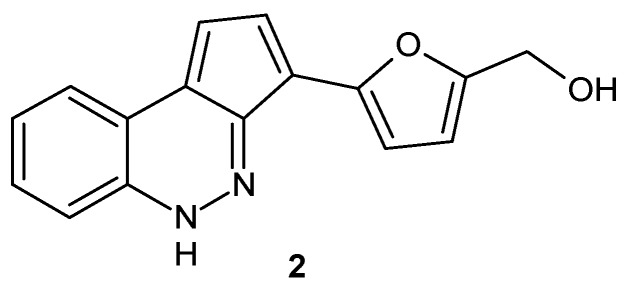
Structure of 2-furanmethanol-(5′→11)-1,3-cyclopentadiene-[5,4-*c*]-1*H*-cinnoline.

Synthesis of cinnoline and its derivatives has been extensively discussed in many papers [2,3,4,5,6,7,8,9]. Until 2011, no compounds containing the cinnoline ring system were found in nature. The first natural cinnoline derivative 2-furanmethanol-(5′→11)-1,3-cyclopentadiene-[5,4-*c*]-1*H*-cinnoline **2** (Figure 2) was isolated from *Cichorium endivia* when investigating the in vitro and in vivo hepatoprotective properties of *Cichorium endivia* L. extract (CEE) [10]. Synthetic molecules bearing a cinnoline framework are extensively studied due to their various biological activities depending on the nature and position of their substituents. In addition, they are often designed as analogs of previously obtained quinoline or isoquinoline derivatives [11,12,13,14]. 

Cinnoline, together with other bicyclic scaffolds, is the subject of our special interest as the terminal moiety of symmetrical compounds designed in agreement with the bisintercalators’ structural requirements [15,16]. Our previous review describing the biological properties of cinnoline derivatives included papers and patents published until 2004 [1]. Herein, we aimed to review documents published from 2005 to 2019, focusing on the compounds bearing a cinnoline nucleus, in particular with respect to their biological activity and potential therapeutic use.

## 2. Biological Activity of Cinnoline Derivatives

### 2.1. Antimicrobial Activity

**Figure 3:**
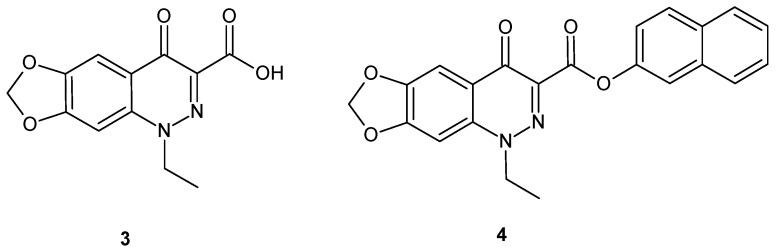
Cinoxacin and its naphthyl ester derivative.

Infectious diseases constitute a growing therapeutic challenge worldwide due to the developing resistance of pathogens to known drugs [17,18]. As a consequence, there is an urgent need to design new compounds with improved activity against drug-sensitive as well as drug-resistant pathogens. Cinnoline derivatives were widely studied as antimicrobial agents [1]. Cinoxacin **3** (Figure 3) is a common drug used in urinary tract infections [19]. Since it has a high phototoxicity index, Vargas et al. synthesized the naphthyl ester of cinoxacin **4** (Figure 3) in order to evaluate its possible application in antibacterial phototherapy. The ester derivative of cinoxacin **4** exhibited comparable photostability and antibacterial activity against *E. coli* to the parent drug but enhanced antibacterial activity upon irradiation [20].

**Figure 4:**
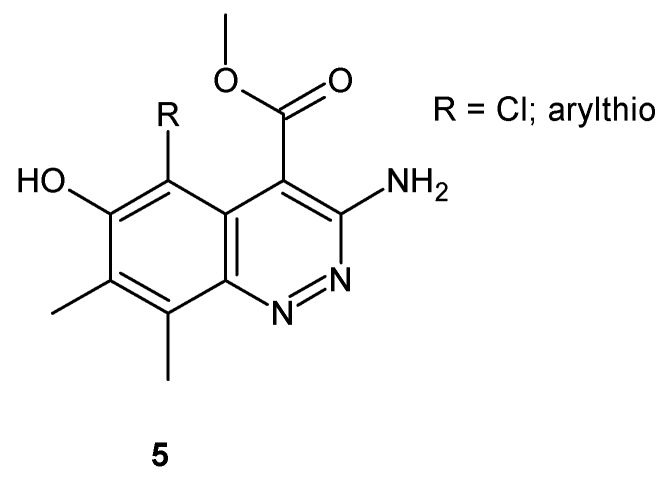
General structure of 6-hydroxycinnoline derivatives.

Depicted in Figure 4, 6-hydroxycinnolines were synthesized and tested for in vitro antifungal activity against *Candida* and *Aspergillus* species. It was elucidated that most of the obtained compounds exhibited potent antifungal activity against *C. krusei*, *C. neoformans*, and *A. niger*, with the highest activity towards *C. neoformans* [21].

**Figure 5:**
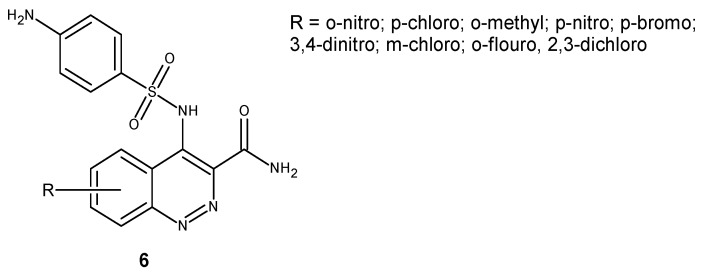
Cinnolines bearing a sulphonamide moiety with antibacterial and antifungal activity.

Cinnoline derivatives bearing sulphonamide moiety **6** (Figure 5) were synthesized as potential antimicrobial and antifungal agents. Evaluation of their activity against a panel of bacteria strains including *P. aeruginosa, E. coli, B. subtilis, S. aureus* and fungi *C. albicans* and *A. niger* revealed that the combination of two active moieties in one molecule resulted in significant activity improvement. Halogen substituted derivatives showed potent activity at lesser concentrations with approximately the same zone of inhibition as the reference drug [22].

**Figure 6:**
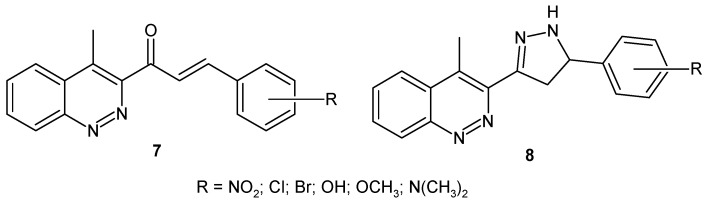
General structures of cinnoline based chalcones and cinnoline based pyrazoline derivatives.

Some new cinnoline based chalcones **7** and cinnoline based pyrazoline derivatives **8** (Figure 6) were evaluated for their antibacterial activity against *B. subtilis*, *E. coli*, *S. aureus* and *K. pneumoniae*, antifungal activity against *A. flavus*, *F. oxysporum*, *A. niger* and *T. viridae* and insecticidal activity against *Periplaneta americana*. The most potent tested compounds were 4-Cl-, 2-NO_2_-, 4-NO_2_-substituted cinnoline based chalcones as well as 3-Cl-, 2-NO_2_- and 4-OH-substituted cinnoline based pyrazolines. In addition, all chloro-substituted derivatives of series **7** and hydroxy-substituted derivatives of series **8** exhibited better insecticidal activity in comparison to the standard drug [23].

**Figure 7:**
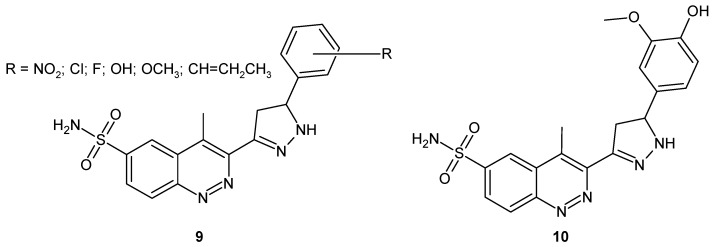
General structure of pyrazole based cinnoline-6-sulphonamides.

In the search for potent antibacterial and antimalarial drugs, Unnissa and co-workers synthesized pyrazole based cinnoline derivatives **9** (Figure 7). All compounds demonstrated significant antitubercular and antifungal activity. Compound **10** 4-methyl-3-[5-(4-hydroxy-3-methoxyphenyl)-4,5-dihydro-1*H*-pyrazol-3-yl]cinnoline-6-sulphonamide (Figure 7) was found to be the most potent with promising activity against resistant strains of *M. tuberculosis* and various pathogenic fungi [24], as well as against protozoan parasite *P. falciparum* [25].

**Figure 8:**
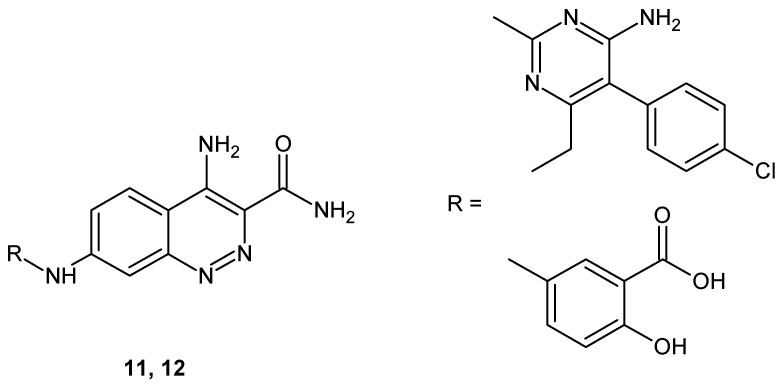
Selected 4-aminocinnoline-3-carboxamide derivatives with antibacterial and antifungal activity.

Parasuraman et al. described 7-substituted 4-aminocinnoline-3-carboxamide derivatives that were evaluated against a panel of Gram+ and Gram− bacteria. All the synthesized compounds exhibited moderate to good antibacterial activity. The MIC (Minimal inhibitory concentration) of tested compounds against *V. cholera, E. coli, B. substills, B. linctus, M. luteus, S. aureus, K. pneumoniae, Corynebacterium* and *S. albus* was found to be in the range of 6.25–25 μg/mL. The most active compounds, **11** and **12** (Figure 8), demonstrated larger or approximately the same zone of inhibition as the reference drug ciprofloxacin. In addition, the synthesized compounds exhibited moderate to good antifungal activity against *A. fumigatus, S. griseus, A. niger, A. parasitus, C. albicans* and *M. ruber*, with the zone of inhibition between 8–27 mm. MIC values were found to be in the range of 6.25–25 μg/mL [26].

**Figure 9:**
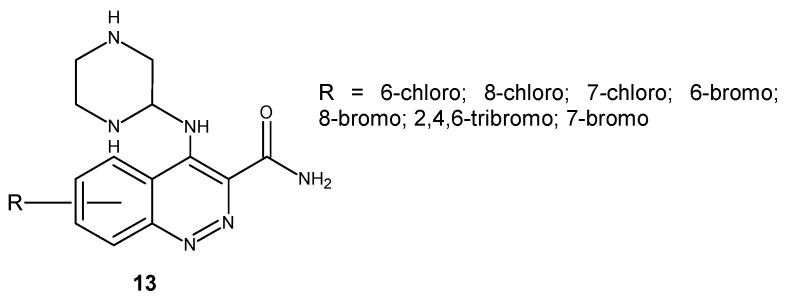
General structure of 4-(*p*-aminopiperazine)cinnoline-3-carboxamide derivatives.

Saxena and co-workers obtained a series of substituted 4-(*p*-aminopiperazine)cinnoline-3-carboxamide derivatives **13** (Figure 9). The in vitro antimicrobial screening against G+ *B. subtilis* and *S. aureus* and G− *E. coli* and *P. aeruginosa* revealed the MIC of the synthesized compounds in the range of 12.5–50 μg/mL, whereas the zone of inhibition was between 6–29 mm. *A. niger* and *C. albicans* were used for evaluation of the antifungal activity. The MIC of the tested compounds was found to be in the range of 12.5–50 μg/mL, whereas the zone of inhibition was between 8–25 mm. The most potent antimicrobial agents in comparison to standard drugs were 6-chloro, 7-chloro and 7-bromo substituted derivatives [27]. 

**Figure 10:**
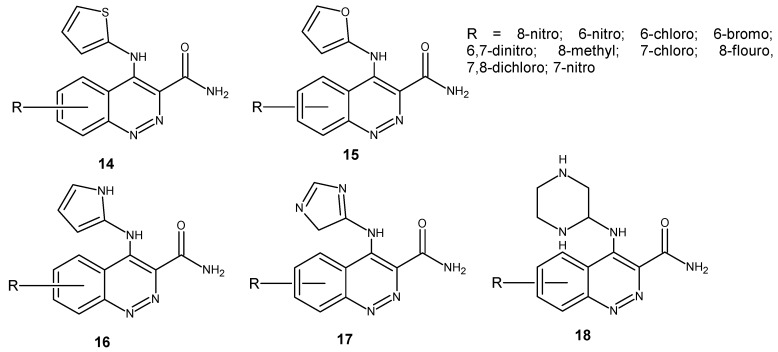
Structure of 4-aminocinnoline-3-carboxamides substituted with five- or six-membered heterocycles.

As a continuation of previous studies, they obtained a new series of cinnoline-3-carboxamide derivatives with five-membered (thiophene **14**, furan **15**, pyrazole **16**, imidazole **17**) or six-membered heterocycle (piperazine **18**) substitutions at the 4-amino group of cinnoline core (Figure 10). Compounds were evaluated for antibacterial, antifungal and anti-inflammatory activity. They exhibited antibacterial activity against *B. subtilis, S. aureus, E. coli* and *P. aeruginosa.* However, the potency of tested compounds differed depending on the substituent at the cinnoline nucleus. The most potent compounds in comparison to the standard drug norfloxacin were 6-chloro substituted compounds. Antifungal activity against *C. albicans* and *A. niger* was observed for all series, but the most potent antifungal agents were the 7-chloro substituted cinnoline thiophene derivative and the 6-chloro substituted cinnoline furan derivative. In all five series, halogen substituted compounds were found to be the most active, followed by methyl substituted and nitro substituted derivatives [28].

**Figure 11:**
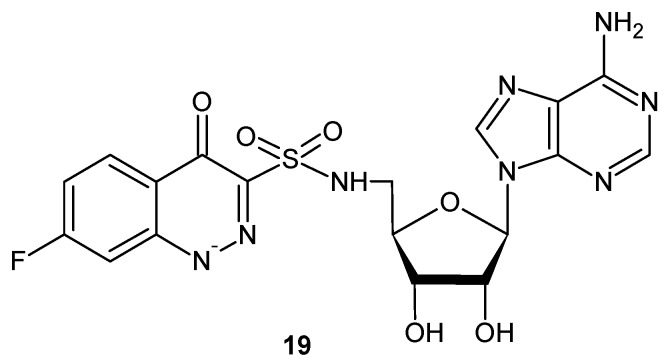
Cinnoline nucleoside analog acting as a siderophore biosynthesis inhibitor.

Looking for potent antitubercular compounds, Dawadi et al. obtained analogues of nucleoside antibiotics where the salicyl-sulfamate moiety was replaced by a cinnolinone-3-sulphonamide group. The most active compound **19** (Figure 11) demonstrated low nanomolar mycobacterial salicylate ligase (MbtA) inhibition and exhibited very good antimycobacterial activity under iron-deficient conditions (MIC = 2.3 µM) by blocking production of siderophores in whole *M. tuberculosis* cells [29].

**Figure 12:**
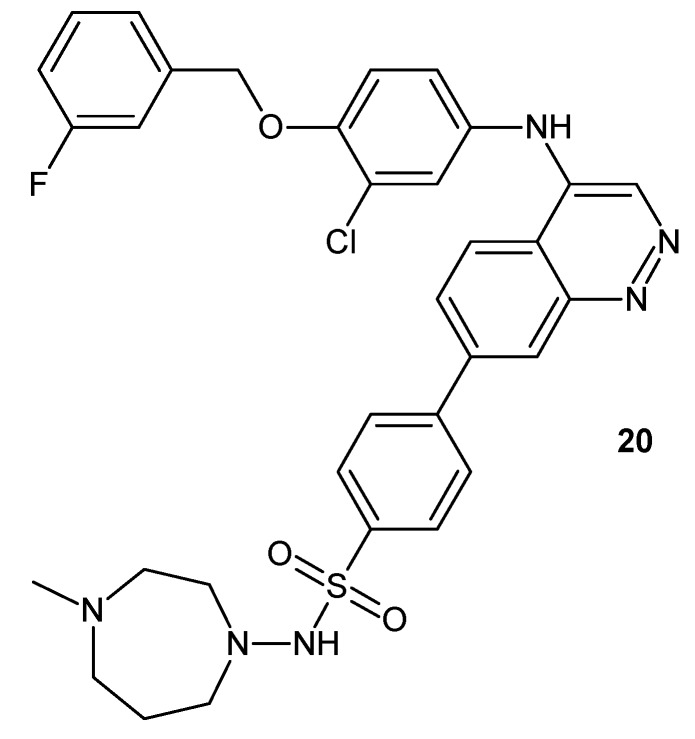
N-(3-chloro-4-((3-fluorobenzyl)oxy)phenyl)-7-(4-((4-methyl-1,4-diazepan-1-yl)sulfonyl)phenyl)cinnolin-4-amine (NEU-1017).

The cinnoline ring system was also used in designing compounds active against tropical protozoan infections. Devine and co-workers synthesized a panel of compounds with different heterocyclic scaffolds (quinoline, isoquinoline, cinnoline, phthalazine, 3-cyanoquinoline). Cinnoline derivative **20** (Figure 12) displayed potent proliferation inhibition for *L. major* and *P. falciparum* (Half maximal effective concentration EC_50_ value = 0.24 µM and 0.003 µM, respectively). In addition, the cinnoline derivative exhibited increased potency against amastigotes (0.24 μM) but with a significant decrease in potency against the promastigote form [12].

**Figure 13:**
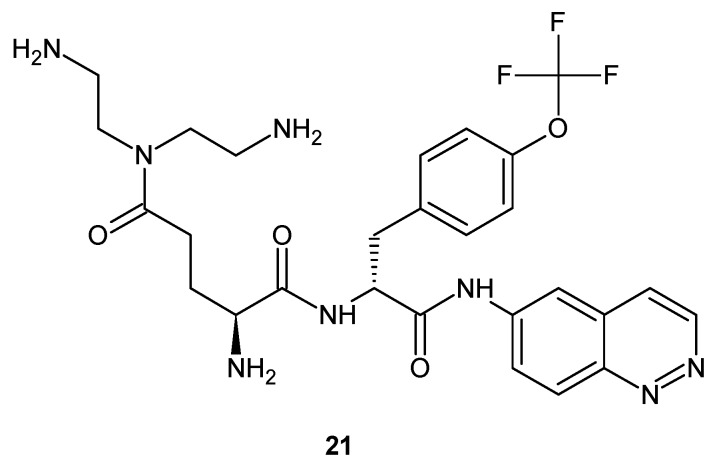
Example of a cinnoline derivative with polybasic functionalities patented as an efflux pump inhibitor.

Some cinnoline derivatives **21** (Figure 13) were patented as compounds active against resistance developing bacteria. Glinka and co-workers described the invention related to efflux pump inhibitor (EPI) compounds having polybasic functionalities. The compounds inhibited bacterial efflux pumps and could be used in combination with an antibacterial agent to treat or prevent bacterial infections. [30].

### 2.2. Analgesic and Antiinflamatory Activities

**Figure 14:**
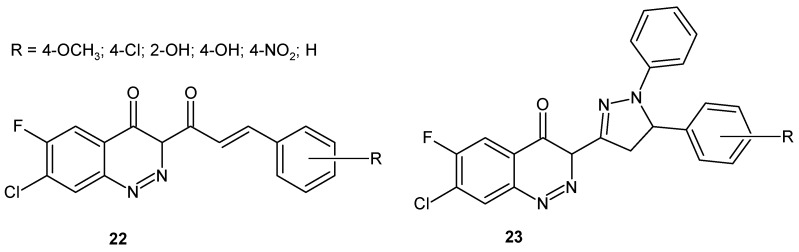
Cinnoline derivatives with dual anti-inflammatory and antibacterial activity.

In an effort to find dual acting compounds, Chaudhary et al. designed a series of cinnoline derivatives with pyrazoline **23** or without a pyrazoline nucleus **22** (Figure 14) as anti-inflammatory and antibacterial agents. It has been shown that cinnolines bearing pyrazoline ring **23** (Figure 14) and having electron donating functional groups at the phenyl moiety (methoxyl and hydroxyl) exhibited the highest anti-inflammatory activity. In case of antibacterial activity, an electron withdrawing substituent at the phenyl group of cinnoline derivatives without pyrazoline ring **22** (Figure 14), as well as hydroxyl substitution of the phenyl ring of cinnoline derivatives with a pyrazoline moiety, were associated with increased activity against G+ (*S. aureus*, *B. subtilis*) and G− bacteria (*E. coli*) [31].

**Figure 15:**
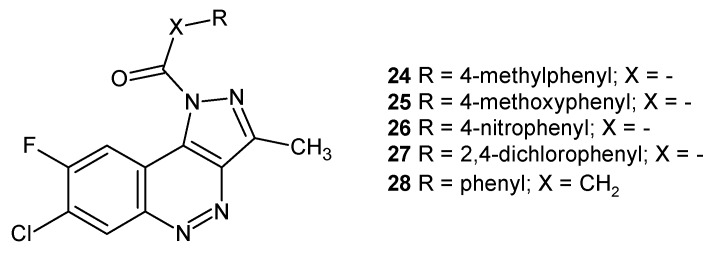
Selected pyrazolo[4,3-*c*]-cinnoline derivatives.

A series of dual acting pyrazolo[4,3-*c*]cinnoline derivatives were also obtained by Tonk and co-workers. It was elucidated that compounds with an electron donating group in the benzoyl ring exhibited higher anti-inflammatory activity than compounds with a benzoyl ring substituted by electron withdrawing groups. Moreover, a methylene spacer between the phenyl group and the carbonyl carbon increased anti-inflammatory activity, whereas the O-CH_2_ group caused a considerable decrease in activity. Compounds that exhibited excellent protection against inflammation **24** and **25,** depicted in Figure 15, also showed a strong cyclooxygenase-2 (COX-2) binding profile. They were considered safer in terms of gastric ulcerogenicity and lipid peroxidation activity than the standard drug naproxen. In case of antibacterial activity, compounds with a 4-nitro- (**26**) or 2,4-dichloro (**27**) substituent at the benzoyl group exhibited significant activity against G− (*E. coli* and *P. aeruginosa*) and G+ (*S. aureus*) bacterial strains. However, compounds with an unsubstituted phenyl ring and methylene spacer **28** (Figure 15) were found to be the best dual anti-inflammatory and antibacterial agent (with significant activity against all three strains) [32].

**Figure 16:**
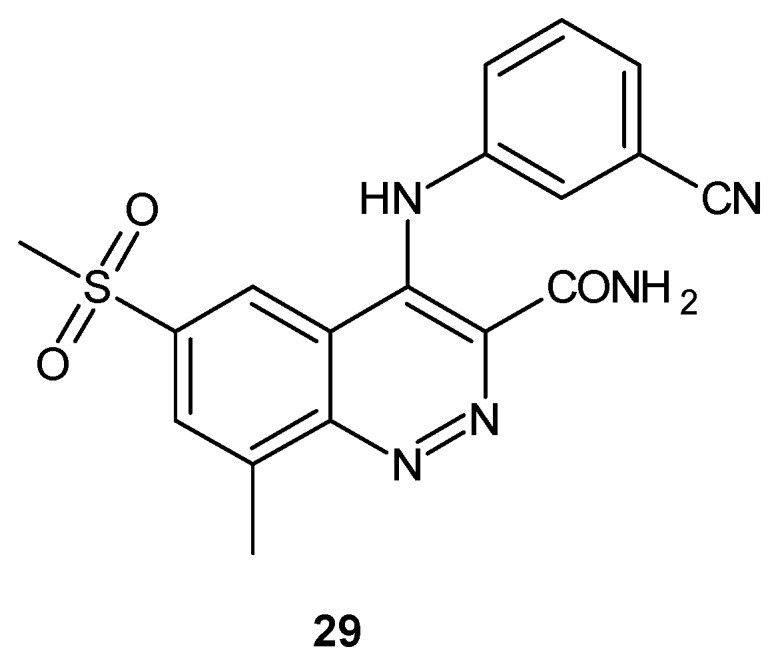
The most promising phosphodiesterase 4 (PDE4) inhibitor with a cinnoline nucleus.

Cinnoline derivatives have been also reported as phosphodiesterase 4 (PDE4) inhibitors [33]. PDE4 is the predominant isoenzyme in almost all immune and inflammatory cells and is an important regulator of cyclic adenosine monophosphate (cAMP) content in airway smooth muscle. Inhibition of PDE4 leads to bronchodilation and the reduction in the production of inflammatory mediators such as tumor necrosis factor (TNF-α) by cAMP down regulation. A PDE4 inhibitor could be used as a potential anti-inflammatory agent in chronic obstructive pulmonary disease (COPD), asthma, rhinitis and rheumatoid arthritis [34]. Structurally related to quinoline PDE4 inhibitors, 3-amido-4-anilinocinnoline **29** has been designed by Lunniss et al. in order to overcome the poor pharmacokinetic profile in the cynomolgus monkey [33]. Compound **29** (Figure 16) retained excellent in vitro potency and >100-fold selectivity versus other PDE isoenzymes with improved pharmacokinetics in the monkey in comparison to the quinoline analog [33].

**Figure 17:**
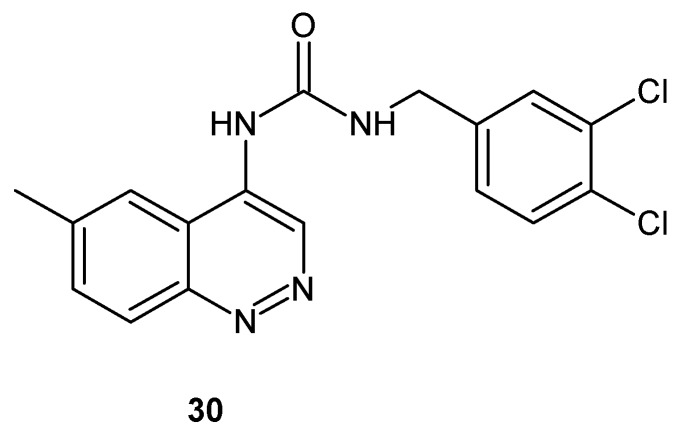
Urea derivative bearing a cinnoline nucleus evaluated as Vanilloid receptor subtype VR1 (TRPV1) receptor antagonist.

Vanilloid receptor subtype VR1 (TRPV1) present in various brain regions, the spinal cord, peripheral sensory neurons and non-neuronal tissues is considered as a new target for pain management but all natural vanilloid receptor agonists such as capsaicin cause an initial burning effect. TRPV1 competitive antagonists, which lack excitatory effects, were designed and evaluated in vivo in animal pain models. Urea derivative bearing cinnoline group **30** (Figure 17) was synthesized among other compounds with various bicyclic heteroaromatic pharmacophores as novel potential analgesics acting through the TRPV1 receptor antagonism ([35] and references therein).

**Figure 18:**
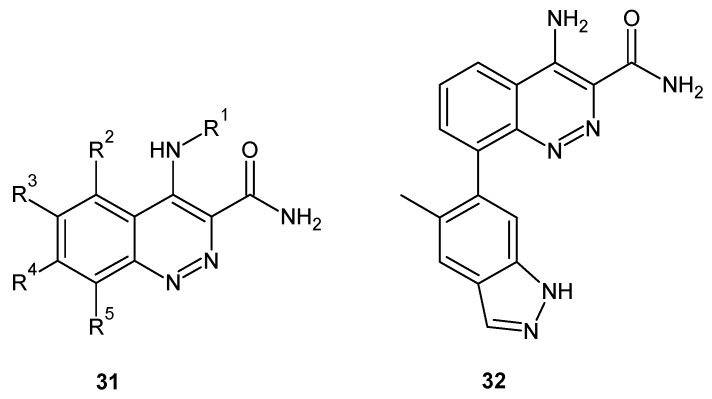
Structure of 4-aminocinnoline-3-carboxamide derivatives that exhibit Bruton’s tyrosine kinase (BTK) inhibition activity.

Since Bruton’s tyrosine kinase (BTK) is a kinase implicated in autoimmune disorders, BTK inhibition is considered as an attractive approach for the treatment of autoimmune diseases such as rheumatoid arthritis [36]. In 2013, scientists from Takeda Pharmaceutical Company Ltd. patented cinnoline derivatives of general formula **31** depicted in Figure 18 as BTK inhibitors [37]. In addition, the discovery of a series of 4-aminocinnoline-3-carboxamides that exhibited BTK inhibition were reported by Smith et al. A fragment-based screening approach incorporating X-ray co-crystallography was used to identify a cinnoline fragment and characterize its binding mode. Optimization of the fragment hit resulted in the identification of compound **32** (Figure 18), an orally absorbed, noncovalent BTK inhibitor reducing paw swelling in a dose- and exposure-dependent fashion in a rat model of collagen-induced arthritis [38].

**Figure 19:**
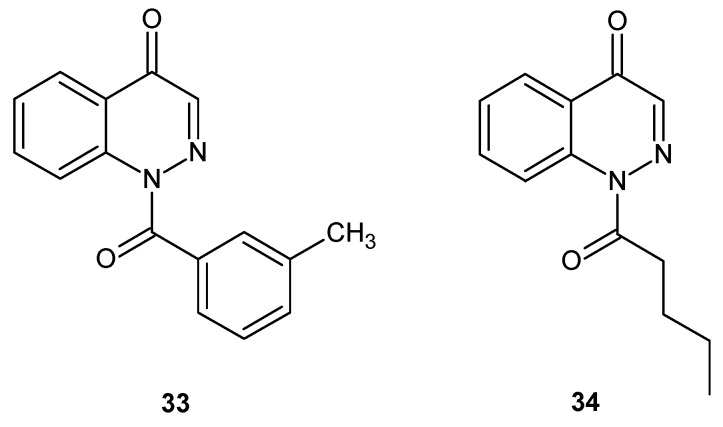
The most potent cinnoline human neutrophil elastase (HNE) reversible competitive inhibitors.

Cinnoline derivatives were also evaluated as human neutrophil elastase (HNE) inhibitors. Excessive HNE activity is connected with many inflammatory disorders and compounds which are able to inhibit the proteolytic activity of HNE represent promising therapeutic agents for the treatment of diseases involving its excessive activity. Potential HNE inhibitors bearing cinnoline scaffolds were designed by transformation of indazole into the cinnoline by enlargement of the pyrazole ring of the *N*-benzoylindazoles reported earlier [39,40]. Studies revealed that although cinnoline derivatives (**33** and **34** were the most potent) (Figure 19) were reversible competitive inhibitors of HNE with increased stability in aqueous solution, they exhibited lower potency in comparison to *N*-benzoylindazoles ([41] and references therein).

**Figure 20:**
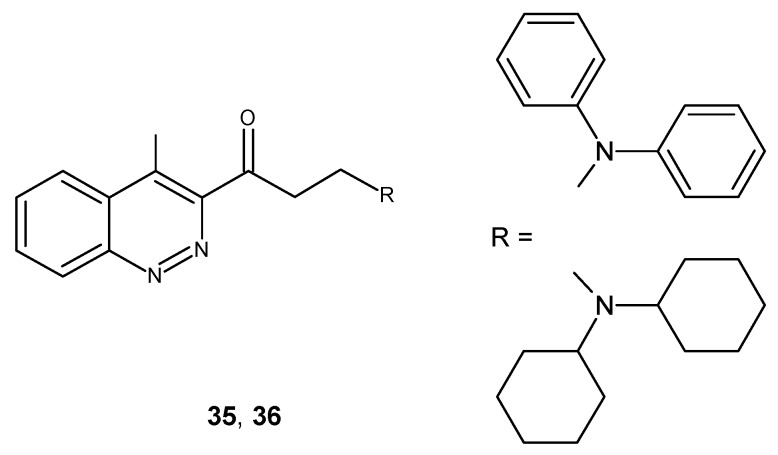
Cinnoline fused Mannich base derivatives.

A cinnoline fused Mannich base with a large hydrophobic diphenyl substituent at amino group **35** (Figure 20) exhibited higher analgesic activity when compared to diclofenac at 120 min and 180 min. In addition, its dose level (50 mg/kg) resulted in similar anti-inflammatory activity in comparison to celecoxib (20 mg/kg). What is more, compound **35** as well as **36** (with a dicyclohexane moiety) (Figure 20) also exhibited antibacterial activity with a larger zone of inhibition when compared to streptomycin in *S. aureus* and *E. coli*, respectively [42].

### 2.3. Potential for Neurological Disorders

Compounds bearing a cinnoline nucleus fused with various heterocyclic scaffolds were also designed as potential therapeutic agents aiming at treating many neurological and psychiatric disorders e.g., Huntington’s [43] or Alzheimer’s disease [44].

**Figure 21:**
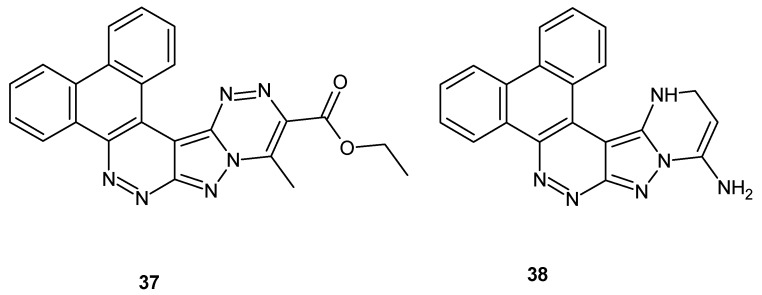
Dibenzopyrazolocinnolines with antiparkinsonian activity.

Amer et al. synthesized dibenzopyrazolocinnolines and evaluated their antiparkinsonian activity. The pharmacological screening revealed that the most active compounds **37** and **38,** depicted in Figure 21, exhibited antiparkinsonian activity comparable to benzatropine [45].

**Figure 22:**
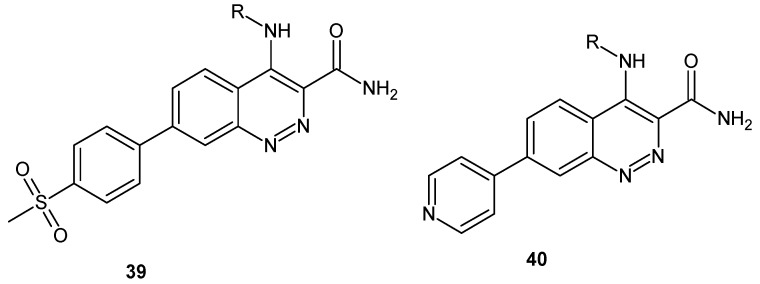
General structures of cinnoline leucine-rich repeat kinase 2 (LRRK2) inhibitors.

Mutations in the leucine-rich repeat kinase 2 (LRRK2) protein have been associated with Parkinson’s disease. Inhibition of LRRK2 kinase activity by a selective small-molecule inhibitor has been proposed as a potential treatment for this disease [46]. Scientists from Elan Pharmaceuticals worked on a series of cinnoline LRRK2 small-molecule inhibitors identified from a kinase-focused high throughput screening (HTS) of an in-house library [47]. In addition, Garofalo et al. reported 4-aminocinnoline-3-carboxamide derivatives **39**, **40** (Figure 22) potent against both wild-type and mutant LRRK2 kinase activity in biochemical and cellular assays. In addition, these compounds exhibited excellent central nervous system penetration. Unfortunately, due to disappointing kinase specificity, they were no longer studied [48].

The phosphodiesterase 10A (PDE10A) enzyme is involved in cellular signaling pathways in schizophrenia. As a consequence, inhibitors of PDE10A offer a promising therapeutic approach for the treatment or prevention of psychiatric disorders, especially schizophrenia and related diseases [49].

**Figure 23:**
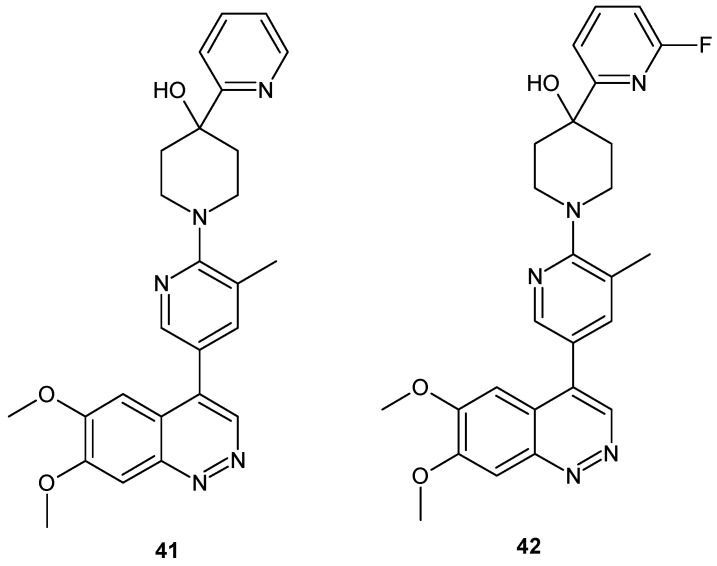
Structure of 6,7-dimethoxy-4-(pyridine-3-yl)cinnolines with potent phosphodiesterase 10A (PDE10A) inhibitory activity.

Hu et al. described 6,7-dimethoxy-4-(pyridine-3-yl)cinnolines as novel phosphodiesterase 10A inhibitors. The mode of binding in the enzyme’s catalytic domain was also elucidated. Selective inhibitor of PDE10A **41** (Figure 23) was selected. It demonstrated efficacy in a rodent behavioral model of schizophrenia and good in vivo metabolic stability in rats [50]. Yang et al. described high in vitro potency of compounds **41** and **42** (Figure 23) for PDE10A with the half maximal inhibitory concentration (IC_50_) values of 1.52 ± 0.18 nM and 2.86 ± 0.10 nM, respectively and 1000-fold selectivity over PDE3A/B and PDE4A/B. These compounds were also suitable for positron emission tomography (PET) radionuclide labelling [51].

**Figure 24:**
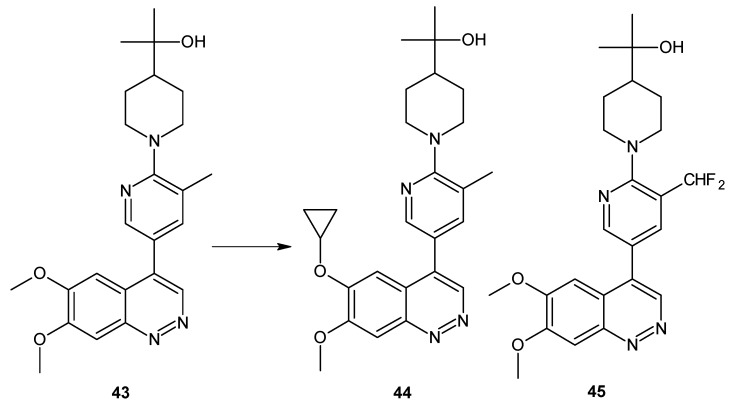
PDE10A inhibitors with improved selectivity.

Since some 6,7-dimethoxy-4-(pyridine-3-yl)cinnolines also exhibited PDE3 activity (a risk of off-target effects), optimization of structure **43** (Figure 24) led to the discovery of compounds **44** and **45** (Figure 24) with significantly improved selectivity against PDE3 but maintaining their PDE10A inhibitory activity and in vivo metabolic stability comparable to **43** (Figure 24) [52].

**Figure 25:**
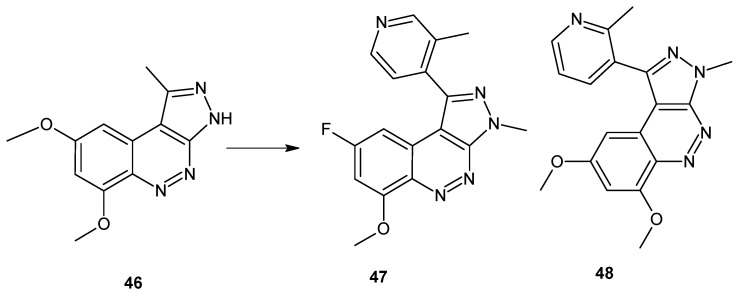
Selected 3*H*-pyrazolo[3,4*-c*]cinnolines that act as potent, selective and brain-penetrant PDE10A inhibitors.

Recently, Geneste et al. described the optimization of HTS hit structure **46** (Figure 25) supported by X-ray crystal structure analysis and molecular modeling which gave 3*H*-pyrazolo[3,4-*c*]cinnolines **47** and **48** (Figure 25), which are potent, selective and brain-penetrant PDE10A inhibitors with an improved pharmacokinetic profile in rats [53]. Preparation of 3*H*-pyrazolo[3,4-*c*]cinnoline derivatives as PDE10A inhibitors was also the subject of patent WO2014/027078 [54].

**Figure 26:**
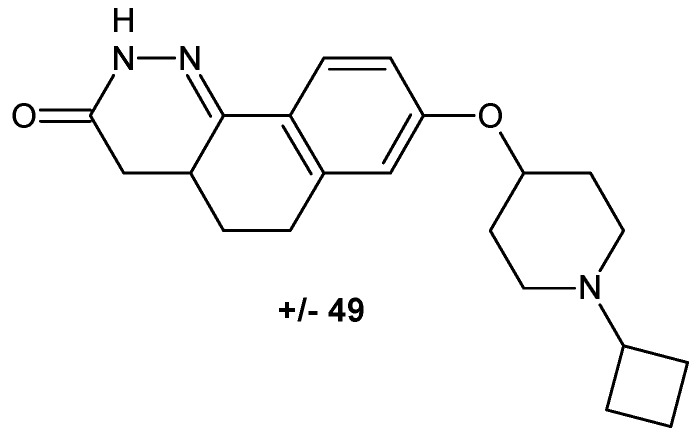
Potent benzocinnolinone analogue of irdabisant with high histamine receptor H_3_ H_3_R binding affinity.

The cinnoline scaffold turned out to be a useful building block in designing compounds targeting histamine receptor H_3_. Involvement of the H_3_ receptor subtype in the presynaptic regulation of the release of various neurotransmitters in the central nervous system makes it an attractive target for treating diseases such as attention-deficit hyperactivity disorder, Alzheimer’s disease, mild cognitive impairment and schizophrenia. Josef and co-workers obtained compounds with the tricyclic benzocinnolinone pyridazinone core as analogues of irdabisant. The compounds 2*H*-benzo[*h*]cinnolin-3-ones and 3*H*-benzo[*f*]cinnolin-2-ones exhibited high H_3_ receptor binding affinity with excellent selectivity against the H_1_R, H_2_R and H_4_R subtypes of histamine receptor. Modification to the linker/amine region of the pharmacophore resulted in **±49** as a mixture of diastereoisomers (Figure 26), which showed improved metabolic stability and rat pharmacokinetics following oral administration ([55] and references therein).

**Figure 27:**
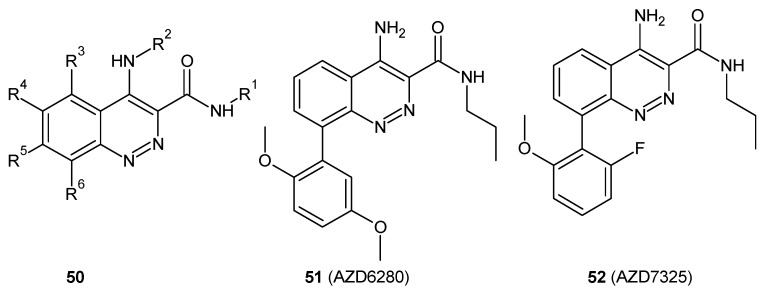
Cinnoline non-benzodiazepine modulators of γ-aminobutyric acid receptor A (GABA A).

Cinnoline derivatives are also enumerated among non-benzodiazepine modulators of γ-aminobutyric acid receptor A (GABA A) [56]. Astra Zeneca works on an orally bioavailable positive modulator of the GABA A α2 and α3 subunits and developed novel compound **50** depicted in Figure 27 as a possible treatment or prophylaxis of anxiety disorders, cognitive disorders, and/or mood disorders [57]. AZD7325 (**51**) and AZD6280 (**52**) depicted in Figure 27 were identified as positive modulators at α2/α3 and negative modulators at α5 GABA A receptors and exhibited a potent anxiolytic-like effect without sedation or cognitive impairment [13,58]. These compounds have undergone clinical trial phase I [59,60]. Moreover, AZD7325 was studied in two phase II proof-of-concept trials in patients with general anxiety disorders (NCT 00807937 and NCT00808249) as well as in a phase II proof-of-mechanism in patients with autism spectrum disorders (NCT01966679). In addition, the diverse metabolite profile of AZD7325 was investigated [61]. In vivo studies in rat and in vitro studies in human, rat, mouse, rabbit and dog liver microsomes were performed with radiolabeled AZD7325, revealing approximately 40 metabolites [61,62].

**Figure 28:**
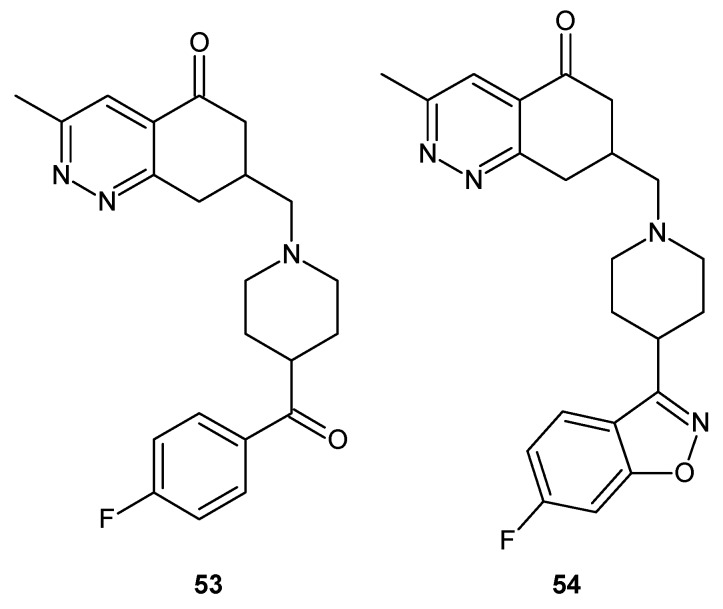
Cinnolinone-diaza analogs of known aminobutyrophenones.

Cinnolinones (**53**, **54**) (Figure 28) as diaza analogues of known aminobutyrophenones were designed as potential atypical psychotics. Determination of the binding affinities towards the serotonin receptors 5-HT_2A_ and 5-HT_2C_, and the dopamine D_2_ receptors revealed that these compounds lacked appreciable affinity for the dopamine D_2_ receptors, and as a consequence, they were not suited as potential psychotics. However, they displayed the highest affinity for the 5-HT_2C_ receptor [63].

### 2.4. Anticancer Properties

**Figure 29:**
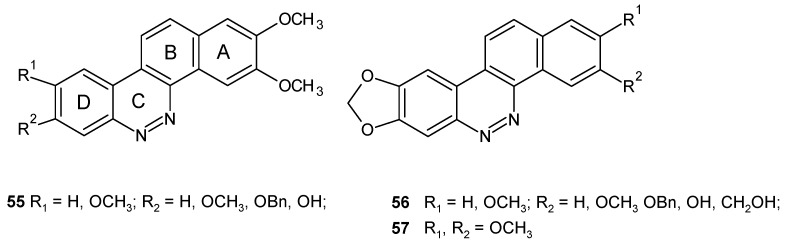
Dibenzo[*c,h*]cinnoline topoisomerase 1 (TOP1) inhibitors.

Cinnoline derivatives were also designed as potential anticancer drugs. Extensive studies have been performed to assess the topoisomerase 1-targeting (TOP1-targeting) activity and cytotoxicity of substituted dibenzo[*c,h*]cinnolines **55**, **56** (Figure 29) as non-CPT (camptothecin) TOP1 inhibitors. Structure-activity relationship (SAR) studies of dibenzo[*c,h*]cinnolines revealed that removal of the methylenedioxy group on the D ring or its replacement by other substituents (methoxy-, benzyloxy- or hydroxy- groups) resulted in a substantial loss of TOP1-targeting activity. The presence of 2,3-dimethoxy substituents in ring A was also determined as a crucial structural element for retaining TOP1 activity and cytotoxicity. Although the substituted dibenzo[*c,h*]cinnolines with significant TOP1-targeting activity exhibited cross-resistance in camptothecin-resistant cell lines, their cytotoxicity was not diminished in cells overexpressing multidrug resistance protein 1 MDR1 [64].

**Figure 30:**
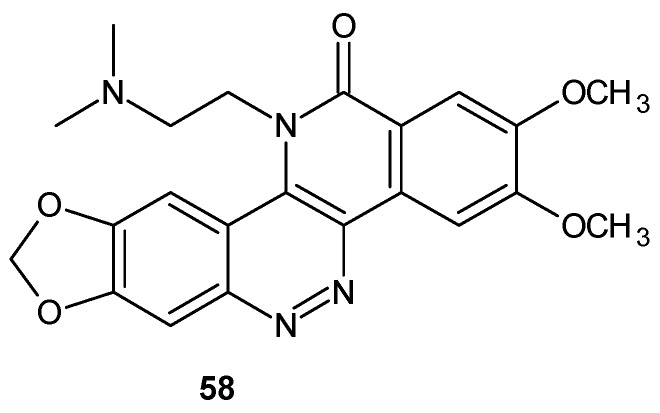
The structure of compound ARC-31.

**Figure 31:**
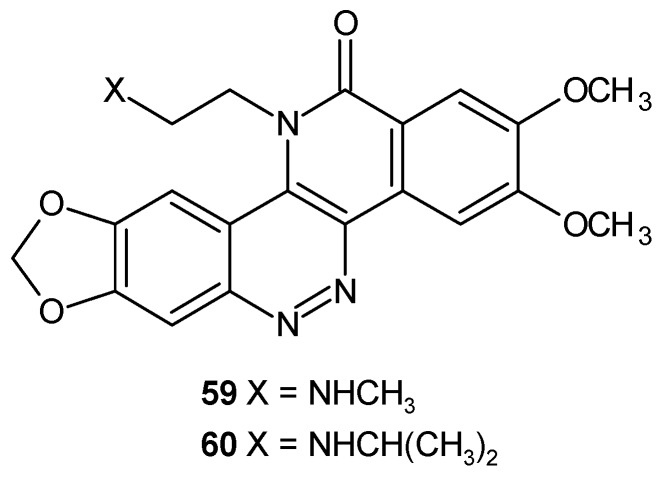
TOP1-acting agents related to ARC-31.

As a continuation of studies on the most potent derivative **57**, 5,6,11-triazachrysen-12-ones with various substituents at 11-position were synthesized [65]. Compound **58** (ARC-31, Figure 30) exhibited an enhanced ability to induce DNA cleavage in the presence of TOP1 and exceptional cytotoxic activity with IC_50_ values below 2 nM against the human lymphoblastoma cell line (RPMI8402) but dose limiting toxicity limited in vivo efficacy in the human tumor xenograft athymic nude mouse model (MDA-MB-435 breast tumor cell line) [66]. In an effort to obtain a less toxic analog with improved efficacy, a number of compounds related to **58** (Figure 30) were synthesized where the 11-ethyl group was substituted at its 2-position with various polar moieties (*N*-methylamino-, *N*-isopropylamino-, hydroxy- and hydroxylamino- groups). These analogs were prepared via the trimethylammonium derivatives of ARC-31 according to methods described in [67]. All analogs exhibited high cytotoxic activity. Although, derivatives with *N*-methylamine **59** and *N*-isopropylamine **60** (Figure 31) exhibited greater cytotoxic activity in vitro in comparison to ARC-31, evaluation in vivo in athymic nude mice showed minimal differences in efficacy in comparison to ARC-31 without therapeutic index improvement [11].

**Figure 32:**
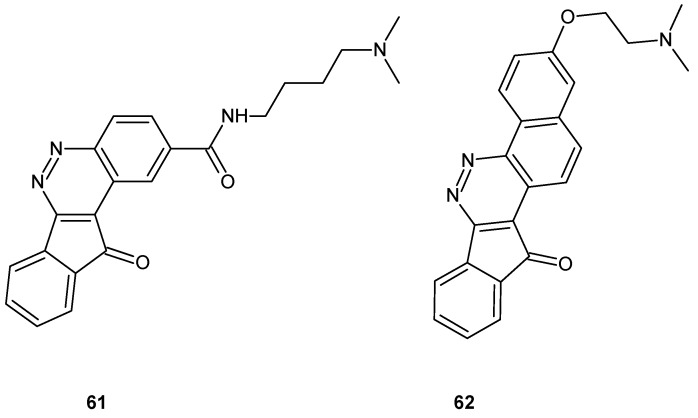
Selected indeno[1,2-*c*]cinnoline) and benzo[*h*]indeno[1,2-*c*]cinnoline derivatives.

Zoidis and co-workers obtained tetra- and pentacyclic cinnoline based compounds indeno[1,2-*c*]cinnoline and benzo[*h*]indeno[1,2-*c*]cinnoline, respectively, bearing protonable amino groups. All tested compounds inhibited proliferation of human cervical carcinoma (HeLa) and human breast adenocarcinoma (MCF-7) cell lines as well as displayed intercalating properties on different nucleic acid strands, with preference for G-quadruplex sequences. The aminobutylamide derivative **61** (Figure 32) exhibited the highest antiproliferative activity with IC_50_ values of 45 nM and 85 nM on HeLa and MCF-7, respectively, whereas the pentacyclic derivative with the same protonable moiety (*N*,*N*-dimethylamine) **62** (Figure 32) caused the highest thermal stabilization in melting studies and exerted acceptable inhibitory activity on human topoisomerase IIα [68].

**Figure 33:**
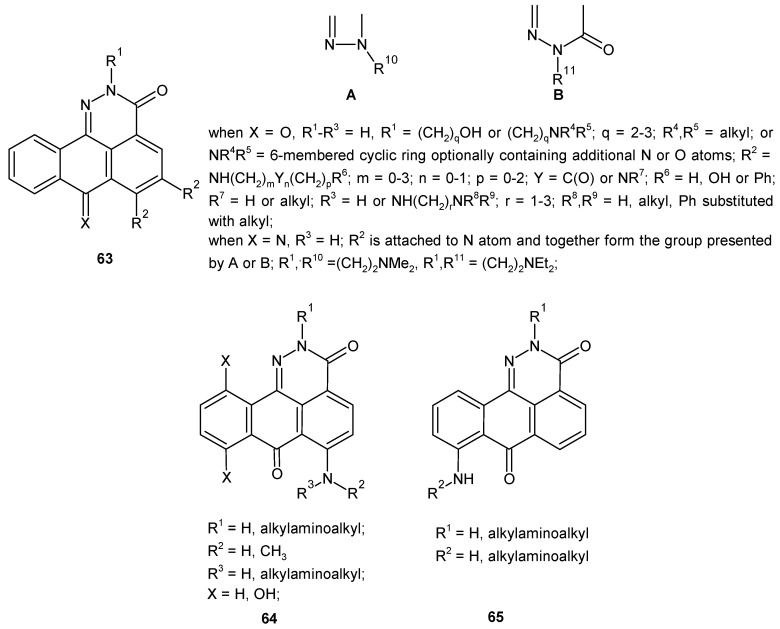
General structure of 2,7-dihydro-3*H*-dibenzo[*de*,*h*]cinnoline-3,7-diones.

Borowski and co-workers obtained a series of anthrapyridazone derivatives **63** (Figure 33) bearing one or two basic side chains at various positions of the tetracyclic core [69,70]. The compounds 2,7-dihydro-3*H*-dibenzo[*de,h*]cinnoline-3,7-diones **64** and **65** (Figure 33) exhibited in vitro cytotoxic activity against murine (L1210) and human (K562) leukemia cell lines. In addition, they were active against human leukemia multi-drug-resistant (K562/DX) cell lines. The most active compounds **64** and **65** (Figure 33) were also tested in vivo against murine P388 leukemia and showed activity comparable to mitoxantrone [71].

**Figure 34:**
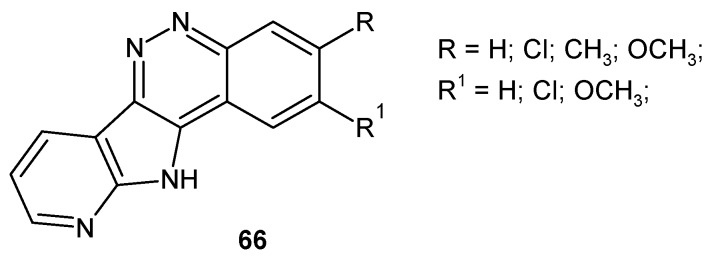
General structure of 11*H*-pyrido[3′,2′:4,5]pyrrolo[3,2*-c*]cinnolines.

Parrino et al. described 11*H*-pyrido[3′,2′:4,5]pyrrolo[3,2-*c*]cinnoline derivative **66** (Figure 34) which exhibited high cytotoxic activity against a panel of 60 human tumor cell lines screened by the National Cancer Institute (Bethesda, MD, USA). Particular efficacy of tested compounds was observed against the leukemia subpanel. In addition, they were also found to be active in cells overexpressing MDR1. The compounds caused apoptosis, mitochondrial depolarization, generation of reactive oxygen species, and the activation of caspase-3, caspase-8, and caspase-9. Moreover, they acted as topoisomerase I inhibitors [72].

**Figure 35:**
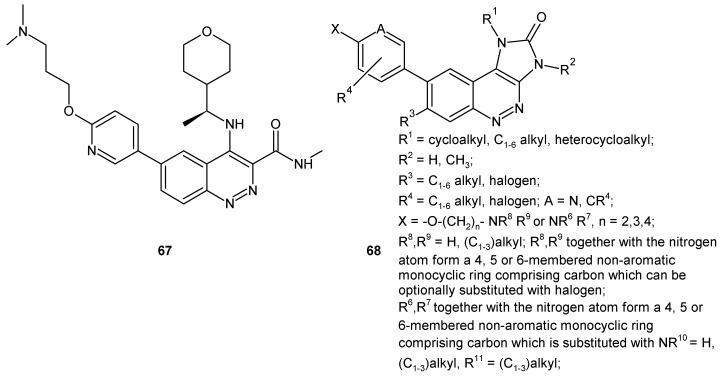
Cinnoline based ataxia teleangiectasia mutated (ATM) inhibitors.

Barlaam and co-workers, while working on the optimization of selective quinoline based inhibitors of ataxia teleangiectasia mutated (ATM) kinase involved in the repair of DNA double strand breaks, synthesized a series of cinnoline-3-carboxamides as suitable replacements of quinoline carboxamides. Compound **67** (Figure 35) was identified as a potent ATM inhibitor with excellent kinase selectivity and good physicochemical and pharmacokinetic properties. Monotherapy with ATM inhibitor **67** did not cause tumor regression in the SW620 colorectal tumor xenograft model, whereas combination with irinotecan resulted in significantly greater tumor growth inhibition in comparison to irinotecan alone [14,73]. The 1,3-dihydroimidazo[4,5-*c*]cinnoline-2-one derivatives of general formula **68** (Figure 35) were patented as ATM modulators used to treat or prevent ATM mediated diseases, including cancer [74].

**Figure 36:**
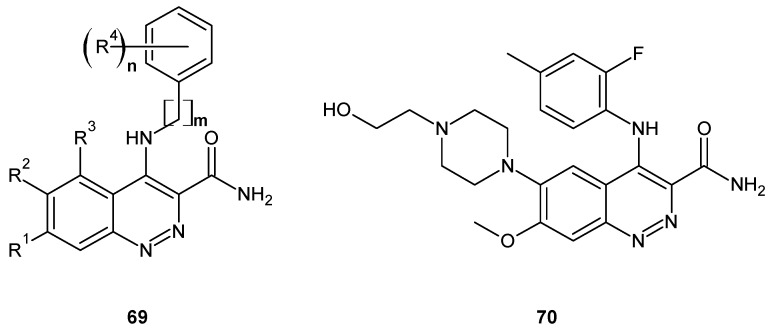
Structure of 3-amido-4-anilinocinnoline derivatives exhibiting colony-stimulating factor-1 receptor (CSF-1R) inhibition.

**Figure 37:**
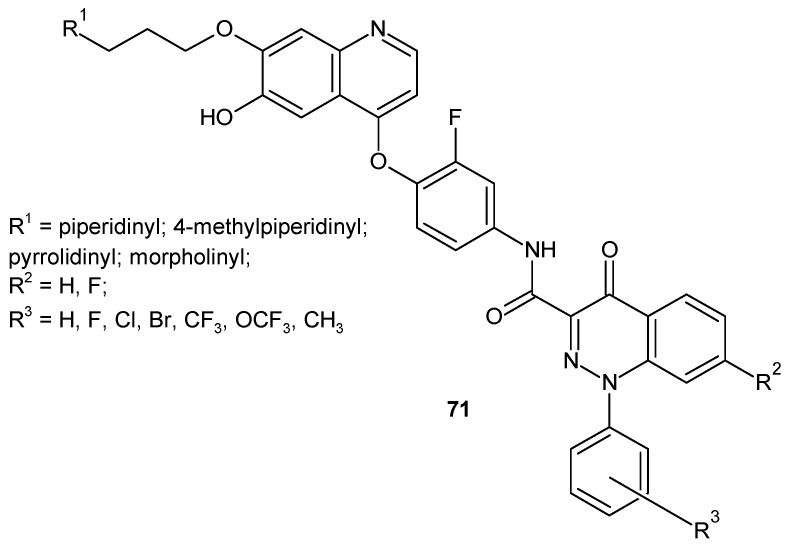
c-Met inhibitor with 4-oxo-1,4-dihydrocinnoline-3-carboxamide moiety.

Colony-stimulating factor-1 (CSF-1) through binding to its receptor (CSF-1R) regulates the migration, proliferation, function, and survival of macrophages [75]. Since CSF-1R is overexpressed in many tumors and at sites of inflammation, CSF-1R inhibitors seem to be an attractive therapeutic strategy for cancer as well as autoimmune and inflammatory diseases. The 3-amido-4-anilinocinnolines of general formula **69** (Figure 36) were reported as potent, highly selective CSF-1R inhibitors [76,77]. They were designed in order to overcome the cardiovascular liability of potent and selective 3-amido-4-anilinoquinoline CSF-1R inhibitor (AZ683), which was able to reduce the level of tumor-associated macrophages in a breast cancer xenograft model. The 3-amido-4-anilinocinnoline compound with 1-hydroxyethylpiperazine substituent at 7 position of cinnoline scaffold **70** (AZD7507, Figure 36) was a potent CSF-1R inhibitor demonstrating good oral pharmacokinetic profile as well as reduced risk of cardiotoxicity in comparison to AZ683 [78].

c-Met receptor tyrosine kinase is another cellular target for compounds designed as potential anticancer agents because it has been found to be overexpressed or mutated in various human cancer cells [79]. Some 4-(2-fluorophenoxy)quinoline derivatives bearing a 4-oxo-1,4-dihydrocinnoline-3-carboxamide moiety **71** (Figure 37) were designed as c-Met inhibitors and evaluated against five c-Met-dependent cancer cell lines and one c-Met-independent cancer cell [80,81]. Most compounds were active against c-Met and the tested cell lines [82].

**Figure 38:**
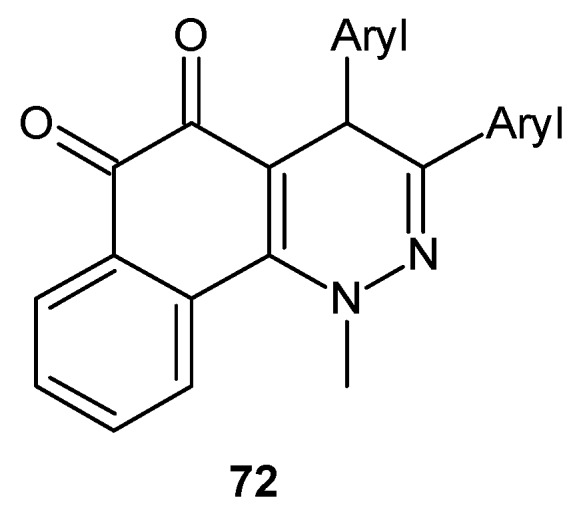
General structure of dihydrobenzo[*h*]cinnoline-5,6-diones.

New dihydrobenzo[*h*]cinnoline-5,6-dione derivatives **72** (Figure 38) were also prepared and evaluated as potential antitumor agents. The majority of tested derivatives exhibited at least moderate cytotoxic activity against a epidermoid carcinoma cell line (KB) and a hepatoma carcinoma cell line (Hep-G2). Nine of the new dihydrobenzo[*h*]cinnoline-5,6-diones displayed a considerable activity profile with IC_50_ values below 5µM against both cell lines. A compound with a 4-NO_2_C_6_H_4_ substituent was identified as the most promising agent with IC_50_ values of 0.56 µM and 0.77 µM against the KB and Hep-G2 cell lines, respectively [83].

**Figure 39:**
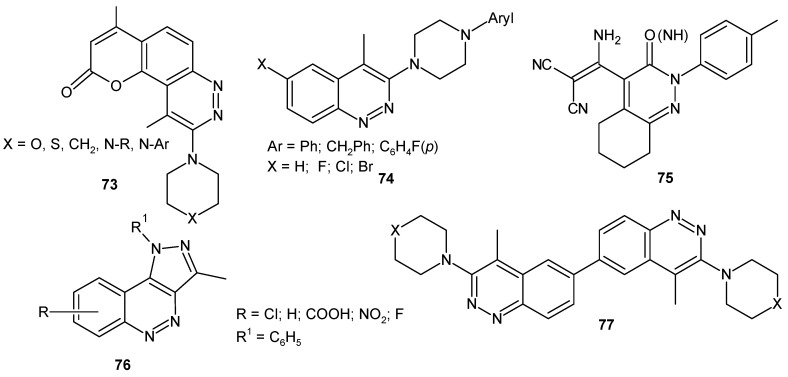
Compounds bearing cinnoline moieties evaluated against breast cancer cell lines.

Many cinnoline derivatives (Figure 39) such as 9-substituted-4,10-dimethylpyrano[2,3-*f*]cinnolin-2-ones with *N*-piperazinyl moieties at C-9 **73 [84]**, 6-substituted-4-methyl-3-(4-arylpiperazin-1-yl)cinnolines **74** [85], hexahydrocinnolines **75** [86] and pyrazolo[4,3-*c*]cinnoline derivatives **76** [87] were synthesized and evaluated in vitro for their antitumor activity against breast cancer cell lines MCF-7 and MDA-231 for bicinnolines **77** [88].

### 2.5. Miscellaneous

**Figure 40:**
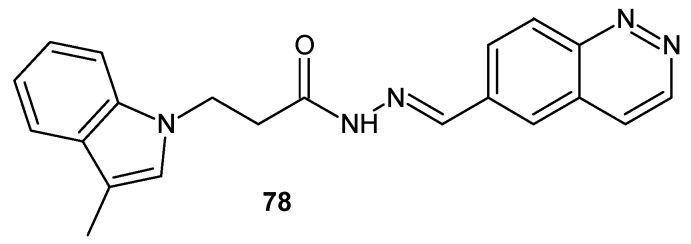
Cinnoline transient receptor potential cation channel, subfamily M, member 5 (TRPM5) inhibitor.

A compound with cinnoline moiety **78** (Figure 40) is patented by Stein and co-authors for very interesting uses. Among other hydrazone derivatives, it was tested as inhibitor of a transient receptor potential cation channel, subfamily M, member 5 (TRPM5) protein which has been shown to be essential for taste transduction. Such compounds could be used as taste inhibitors when administered as a component of pharmaceutical or food products to improve acceptance. Moreover, these agents are intended for use in treating diabetes mellitus, obesity, insulin resistance syndrome and many more [89].

Cinnoline derivatives were also patented as thyroid hormone receptor agonists [90], orexin receptor antagonists [91,92,93,94,95], histone deacetylase (HDAC) inhibitors [96], liver X receptors β selective modulators for the treatment of atherosclerosis [97], somatostatin regulators [98] or cannabinoid-1 receptor inverse agonists [99] and many more.

## 3. Conclusions

In this paper, we have presented a review of studies focused on the biological activity of cinnoline derivatives conducted by many research groups worldwide between 2005 and 2019. The provided information clearly indicates the enormous significance of the cinnoline framework as a building block of many valuable compounds. Compounds bearing the cinnoline scaffold are able to interact with a variety of molecular targets including receptors such as GABA A, CSF-1R, H_3_R and enzymes such as cyclooxygenase-2, topoisomerases, phosphodiesterase, human neutrophil elastase, Bruton’s tyrosine kinase involved in pathogenesis of many diseases. As a consequence, they are intended to be used as antibacterial, antifungal, antimalarial, anti-inflammatory, analgesic, anxiolytic and antitumor agents. Some cinnoline derivatives are under evaluation in clinical trials. There is no doubt that development of cinnoline based molecules constitutes a significant contribution to the identification of lead compounds with optimized pharmacodynamic and pharmacokinetic properties.
